# A Novel Artificial Fish Swarm Algorithm for Recalibration of Fiber Optic Gyroscope Error Parameters

**DOI:** 10.3390/s150510547

**Published:** 2015-05-05

**Authors:** Yanbin Gao, Lianwu Guan, Tingjun Wang, Yunlong Sun

**Affiliations:** 1 Institute of Inertial Navigation and Measurement & Control Technology, College of Automation, Harbin Engineering University, Harbin 150001, China; E-Mails: gaoyanbin@hrbeu.edu.cn (Y.G.); sunyunlong@hrbeu.edu.cn (Y.S.); 2 NavINST-Navigation and Instrumentation Research Group, Department of Electrical and Computer Engineering, Queen’s University, Kingston, ON K7L 3N6, Canada; 3 No. 16 of China Aerospace Science and Technology Corporation, Xi’an 710001, China; E-Mail: wangtingjun0309@126.com

**Keywords:** novel artificial fish swarm algorithm, fiber optic gyroscope, error coefficients recalibration, Monte Carlo simulation, swarm intelligence optimization

## Abstract

The artificial fish swarm algorithm (AFSA) is one of the state-of-the-art swarm intelligent techniques, which is widely utilized for optimization purposes. Fiber optic gyroscope (FOG) error parameters such as scale factors, biases and misalignment errors are relatively unstable, especially with the environmental disturbances and the aging of fiber coils. These uncalibrated error parameters are the main reasons that the precision of FOG-based strapdown inertial navigation system (SINS) degraded. This research is mainly on the application of a novel artificial fish swarm algorithm (NAFSA) on FOG error coefficients recalibration/identification. First, the NAFSA avoided the demerits (e.g., lack of using artificial fishes’ pervious experiences, lack of existing balance between exploration and exploitation, and high computational cost) of the standard AFSA during the optimization process. To solve these weak points, functional behaviors and the overall procedures of AFSA have been improved with some parameters eliminated and several supplementary parameters added. Second, a hybrid FOG error coefficients recalibration algorithm has been proposed based on NAFSA and Monte Carlo simulation (MCS) approaches. This combination leads to maximum utilization of the involved approaches for FOG error coefficients recalibration. After that, the NAFSA is verified with simulation and experiments and its priorities are compared with that of the conventional calibration method and optimal AFSA. Results demonstrate high efficiency of the NAFSA on FOG error coefficients recalibration.

## 1. Introduction

The artificial fish swarm algorithm (AFSA) is one of the state-of-the-art swarm intelligence approaches, which was proposed by Li Xiaolei in 2002 [1]. It is inspired by the autonomous collective movement of artificial fishes (AFs) and their various social behaviors. Its characteristics of global search, quick convergence rate, and efficient search are based on modern elicitation methods [2,3]. After AFSA appeared, it offered new ideas to solve the optimization problems in signal processing [4,5], neural network classifiers [6,7], data mining and clustering [8,9], multi-objective optimization [10,11] and PID controller parameters optimization [12], *etc.*

Nevertheless, the standard AFSA (SAFSA) has not been further considered by researchers, due to its complexity in comparison with other swarm intelligence algorithms in this domain, particularly particle swarm optimization (PSO), whereas the results of SAFSA are not better than those of PSO [13]. PSO is another swarm intelligence algorithm that simulates the natural evolutionary process to solve complex optimization problems. It has been successfully utilized in optimization problems, such as the multidimensional knapsack problem, the economic and economic statistical designs, the complex network reliability problem [14,15,16,17], and so on. However, the reasons for SAFSA’s inefficiency are high structural and computational complexities, lack of using AFs’ previous experiences, lack of appropriate balance between exploration and exploitation to improve the optimization process. Fortunately, the optimal AFSA (OAFSA) with improvement on *Visual* and *Step* parameters to keep a balance between exploration and exploitation, has been utilized in various applications [5,7,18]. More noteworthy is that a novel AFSA (NAFSA) was proposed to conquer all weaknesses of SAFSA and first used for data clustering by Yazdani in 2013 [19]. In NAFSA, different stages of SAFSA are modified to eliminate the demerits, and, thus, improve the efficiency of the algorithm. The modifications include reducing the structural complexity as well as the computational complexity of the algorithm, determining a balance between the exploration and exploitation during the optimization process, and also adopting the AFs’ previous experiences to improve the optimization process. 

On the other hand, the FOG error coefficients recalibration is to identify FOG error parameters accurately after operating for a period of time. It is necessary to recalibrate the FOG error coefficients because they would be slightly changed by environmental disturbances and the aging of the fiber coils [20,21]. Otherwise, the accuracy of FOG-based strapdown inertial navigation system (SINS) would be decreased by these uncalibrated error coefficients [22,23,24]. Therefore, making the recalibration of FOG error coefficients during a specific interval according to FOG’s instability is necessary to maintain the accuracy of FOG-based SINS. However, the conventional expensive and high-precision turntable calibration method and systematic calibration method have the nature of high workload and costs, and the observable characteristic of different parameters is not the same [25]. In addition, its reference information is provided from external equipment so that the calibration precision is dependent on the accuracy of the external equipment [26]. The high workload and costs of conventional calibration method are also not affordable for low costs applications. Therefore, the focus on FOG error parameters’ recalibration to eliminate these drawbacks is always a hot research point.

The OAFSA was utilized for FOG random drift modeling in the navigation applications of AFSA in 2012 by Wang Tingjun [27]. Meanwhile, the OAFSA also used for the real-time ring laser gyroscope bias temperature error compensation in 2014 by Yu Xudong [28]. Moreover, Gao Yanbin has successfully adopted the OAFSA to calibrate the error parameters of FOG and verified the feasibility of OAFSA on FOG error coefficients recalibration [29,30]. However, OAFSA only balanced the exploration and exploitation abilities during the optimization process by the modification on AFs’ *Visual* and *Step* parameters. Additionally, the secondary initialization method after certain times of OAFSA optimization manually increased the non-autonomous property of the OAFSA. But the structural and computational complexities of OAFSA remain and the AFs’ previous experiences are not used for improving the convergence rate. Therefore, solving these issues and letting the NAFSA recalibrate the FOG error coefficients are of great value to improve the overall navigation precision of FOG-based SINS.

The Monte Carlo simulation (MCS) method is a broad class of computational algorithms that relies on repeated random sampling to obtain numerical results [31]. In this research, it is adopted to simulate the NAFSA process for increasing the credibility of FOG error coefficients recalibration results; hence, the computational results are closer to real conditions. Furthermore, it has the priority of reducing workload and costs over conventional expensive and high-precision turntable calibration methods. So the overall advantages of the MCS-NAFSA for FOG error parameters identification are (1) that the algorithm’s structural and computational complexities are reduced to release the high computational cost; (2) that the algorithm’s convergence rate is improved by adopting AFs’ previous experiences during AFs optimization process; (3) that no external reference information is introduced into the identification process; (4) that the high workload and costs in conventional calibration method are decreased greatly; and (5) that the non-autonomous characteristic of OAFSA on FOG error parameters recalibration is avoided. Therefore, the hybrid MCS-NAFSA technique that utilized on FOG error parameters recalibration is the main contribution of this research.

The rest of this paper is organized as follows. In Section 2, the SAFSA and its disadvantages on FOG error parameters recalibration are first presented. Then, the OAFSA and the corresponding secondary initialization method on FOG error parameters recalibration are briefly dedicated. Finally, the NAFSA and its advantages on FOG error parameters recalibration are described with details. Section 3 indicates the FOG error parameters MCS-NAFSA implementation procedures. After that, the MCS-NAFSA FOG error parameters simulation is conducted, and the results are discussed in Section 4. Next, Section 5 demonstrates the FOG-based SINS navigation experiments and discussion with FOG error parameters recalibrated by NAFSA. Section 6 concludes this article.

## 2. Artificial Fish Swarm Algorithm

### 2.1. SAFSA and Its Demerits on FOG Error Coefficients Recalibration

Generally, fish move to the areas that have more food by their individual or swarm search. The AFs model is depicted by prey, swarm, free moving, and following behaviors [1,2,3]. The AFs food consistency degree in specific areas is the AFSA objective function as well as the AFs approach to the maximum food density point. The state of AF *i* is denoted as vector *X* = (*x*_1_, *x*_2_, …, *x_n_*), and *x_i_*(*i* = 1, 2, …, *n*) are the optimization variables. The current food consistency degree of AF *i* in position *X* can be expressed as objective function *Y* = *f*(*X_i_*). *Visual* is the sight field of AFs and *Step* represents the maximum length of each movement. The distance between two AFs in *X_i_* and *X_j_* positions is shown by Euclidean Distance *Dis_i_*_,*j*_ = |*X_i_* − *X_j_*|. Moreover, the best AFs position is loaded in *bulletin* and *crowd factor* δ(0 < δ < 1) represents the AFs crowd degree within its *Visual* range.

According to the characteristics of SAFSA, there are some demerits for its application on the recalibration of FOG error parameters [29,30]. The first one is the AFs lack of the application of previous experiences, which would lead the AFs falling into local extremes during the optimization process. For FOG error parameters identification, this demerit would lead the FOG error parameters to non-optimum values. The second one is that AFs lack the balance between exploration and exploitation during the optimization process, which deteriorates the convergence rate and accuracy of AFs optimization. So the FOG error parameters optimization process would require more time to implement the optimum results. The last demerit is that the structural and computational complexities of SAFSA are high, which will cost more memory loads during optimization process. So it is unsuitable for FOG-based SINS with high real-time computational requirements. Therefore, these demerits should be eliminated before the SAFSA is applied to FOG error coefficients identification.

### 2.2. OAFSA and Its Shortcomings on FOG Error Coefficients Recalibration

Usually, the varied *Visual* and *Step* parameters are used to improve the algorithm’s precision and convergence rate [5,7,18]. Furthermore, the secondary initialization method is also utilized for higher precision FOG error parameters recalibration [29,30]. When the initialization value of parameters *Visual* and *Step* are relatively large, the exploration ability of AFSA is enhanced while the exploitation ability is weakened. Conversely, if the *Visual* and *Step* parameters are relatively small, AFSA’s exploration ability is weakened and the exploitation ability is enhanced. Therefore, a varied *Visual* and *Step* parameters are adopted as [27]:

(1) $$Visual=Visual\cdot \lambda +Visual_{\min}$$

(2) $$Step=Step\cdot \lambda +Step_{\min}$$

(3) $$\lambda =\exp(-3\times (G/G_{\max}))$$

where, *G* and *G*_max_ denote the current iteration times and the preset maximum iteration times, λ denotes the attenuation function, which could balance the exploration and exploitation abilities during the overall optimization process.

Moreover, after dozens of iterations, the indicator function may present a divergence tendency because the AFs fall into the local extreme by unsuccessful prey behavior [29,30]. The former AFs parameters and the optimized FOG error parameters have reached their limits to implement higher precision. In this case, the secondary initialization method is utilized to the AFs swarm and the related FOG error parameters recalibration procedures. Firstly, the variation tendency of indicator function is observed until it presents divergence tendency, and the AFs parameters and FOG error coefficients are stored in the lowest indicator function point. Secondly, the changed AFs parameters are reloaded and the former saved FOG error coefficients are reloaded manually, which is obtained from the former optimization process. Finally, the AFSA optimization process is executed again to reach higher optimization precision.

However, when OAFSA is used for FOG error parameters identification, only the second drawback of SAFSA is eliminated, but the other two drawbacks are not avoided during the optimization process. Moreover, by inducing the secondary initialization method, the lowest indicator function point selection at first stage is artificially aided. The reload process of AFs parameters and FOG error coefficients are completed manually. This means the method is non-autonomous during the optimization process. Therefore, there are also some shortcomings when OAFSA is applied to FOG error parameters identification.

### 2.3. NAFSA and Its Advantages for FOG Error Parameters Recalibration

NAFSA was first proposed and used in data clustering by Yazdani in 2013 [19]. It solved these mentioned disadvantages by improving AFSA’s functional behaviors and overall procedures with some AFs parameters eliminated and several supplementary parameters added. More details on the improvements of AFSA are shown in the following NAFSA parameters and behaviors introduction parts.

#### 2.3.1. Parameters of NAFSA

Suppose there are *N* AFs in D-dimensional space, the position of AF *i* could be denoted as vector *X_i_* = (*x_i_*_,1_, *x_i_*_,2_, …, *x_i_*,*_D_*). AFs *Visual* could be expressed as vector *Visual* = (*v*_1_, *v*_2_, …, *v_D_*), the *Visual* dimensions are determined by the inner coverage of searching space. Therefore, NAFSA could use different *Visual* in various space ranges. The components of vector *Visual* are divided into many parts that make the AFs have better global optimization ability. So NAFSA has higher precision in global extreme optimal ranges. Moreover, the *Contraction Factor* (*CF*) parameter is utilized to substitute *Step* and *crowd factor* parameters. It is introduced to NAFSA for choosing different *Visual* values in different optimal process, and *CF* is an integer less than 1, whether a constant or a function. Previously, the *inertial weight* parameter was applied in PSO for balancing the exploration and exploitation abilities during optimization process [32]. The *CF* in NAFSA has the similar function to *inertial weight* in PSO. Here, random function is adopted to generate *CF* in all iteration process:

(4) $$CF=CF_{\min}+(CF_{\max}-CF_{\min})\times \mathrm{Rand}(0,1)$$

The above equation generates a random *CF* in [*CF*_min_, *CF*_max_]. Therefore, the *i*th element of vector *Visual* in next iteration could be expressed as:

(5) Visual(t+1)=Visual(t)×CF

Next, the NAFSA behaviors will be discussed.

#### 2.3.2. Behaviors of NAFSA

##### Individual Behavior

Individual behavior is made up of prey and free moving behaviors. The AF *i* in position *X_i_*(*t*) tries several times of movement to better position. In each iteration process, AF *i* will occupy the position *X_j_*(*t*) by prey behavior, and then evaluate their food density. If *f*(X*_i_*) ≥ *f*(X*_j_*), then the next position is expressed as:

(6) $$X_{i}(t+1)=X_{j}$$

Because the position *X_j_*(*t*) is within the *Visual* range of AF *i*, the move distance of AF *i* would be less or equal to *Visual* vector in the same dimension. If *f*(X*_i_*) ≥ *f*(X*_j_*), the AF *i* will move to a better position with several iterations by Equation (6) or by prey behavior and Equation (6). However, if *f*(X*_i_*) < *f*(X*_j_*), the AF *i* would not move towards *X_j_*(*t*) and it will find a better position from its previous position. Therefore, for single individual behavior, the AF could find better position by trying several times. Otherwise, if AF *i* could not find a better position after all attempts, the AF could move one *Step* randomly within its *Visual* range:

(7) $$X_{i}(t+1)=X_{i}(t)+Visual\times Rand(-1,1)$$

In NAFSA, each AF moves towards better position by individual behavior. But when an individual fails, it will perform random behavior in its *Visual* range and may discard its previous position, which may find a worse position in the searching space. Nevertheless, in order to keep the AFs swarm diversity and find better position in later optimal behavior, performing the random behavior is necessary for the AFs swarm. Moreover, the AFs position search through random behavior would not be used as best AFs position, so the best AFs position would not be lost even if AFs could not find a better position. In this case, the best AFs position is what has been searched previously. Therefore, in NAFSA, the current AFs position is the best position, so the *bulletin* parameter in SAFSA is no longer a necessity.

##### Group Behavior

Group behavior performs instead of following and swarm behaviors. Keeping all AFs swarm characteristics and making AFs movement in the best position are two main targets in group behavior. The center position of AFs swarm is obtained by swarm behavior. If *f*(*X_Center_*) > *f*(*X_i_*), then the next position of AF *i* is:

(8) $$X_{i}(t+1)=X_{i}(t)+\frac{X_{Center}-X_{i}(t)}{Dis_{i,Center}}\times \left[Visual\times Rand(0,1)\right]$$

If *f*(*X_Center_*) ≤ *f*(*X_i_*), AF *i* could not move towards the center position, while moving towards the best position in the searching space:

(9) $$X_{i}(t+1)=X_{i}(t)+\frac{X_{Best}-X_{i}(t)}{Dis_{i,Best}}\times \left[Visual\times Rand(0,1)\right]$$

Therefore, the AFs in a worse position would move towards center position by comparing with the center position. When the position is better than center position, it will move towards the best AFs swarm position. Therefore, all the AFs will reach the best position by performing group behavior. Consequently, in NAFSA, the best position searched by fish swarm would be adopted to accelerate the convergence rate with all AFs movement. So the group behavior is used to maintain the fish swarm characteristics and avoid reducing swarm diversity.

In group behavior, the center AFs position may have better food density (indicator function) than the best AFs position. AFs move towards center position by Equation (8), but a worse position may exist between the current position and center position. The AFs position may then get worse or even lose their best position by executing Equation (8). Therefore, if the indicator function of center position is better than the best AFs position, the best AFs position is determined by the following equation:

(10) $$X_{Best}(t+1)=X_{Center}$$

The above equation executes only when *f*(*X_Center_*) < *f*(*X_Best_*), while the other AFs movement by Equation (8) helps to maintain the diversity of fish swarm.

#### 2.3.3. Advantages of NAFSA for FOG Error Parameters Recalibration

According to the introduction of NAFSA, there are three main advantages for its applications with FOG error parameters recalibration. The first one is that the parameters reduction and behavioral simplification of NAFSA reduced the structural and computational complexities, which means the NAFSA will cost less memory loads when it is used in FOG error parameters recalibration. The second one is that the choice of *CF* parameter can balance the exploitation and exploration abilities during AFs optimization process. Additionally, *CF* parameters could avoid the local extreme during the AFs optimization process, so the NAFSA could neglect the secondary initialization method in OAFSA and implement the autonomous characteristic when it is adopted in FOG error parameters recalibration. The third one is that the *CF* parameters would reveal the application of AFs previous experiences, which equates to a faster convergence rate during the AFs optimization process. It could implement the FOG error parameters identification with less time and higher precision. Therefore, the NAFSA is more suitable for FOG error parameters recalibration when it is compared with the previous OAFSA.

## 3. FOG Error Coefficients Recalibration by NAFSA

In this section, the tri-axial FOG static error model will be provided at first. And then the optimization indicator function derivation process is presented. Finally, the FOG error coefficients identification procedures by NAFSA will be demonstrated specifically.

### 3.1. FOG Static Error Model

The purpose of error coefficients recalibration is to identify the FOG error parameters accurately, quickly and steadily. There are various recalibration methodologies for FOG error parameters [33,34,35]. The static error model of tri-axial FOG is shown [35]:

(11) $$\left[\begin{array}{c} N_{gx}\\ N_{gy}\\ N_{gz} \end{array}\right]=\left[\begin{array}{ccc} K_{gx} & K_{gx}E_{gxz} & K_{gx}E_{gxy}\\ K_{gy}E_{gyz} & K_{gy} & K_{gy}E_{gyx}\\ K_{gz}E_{gzy} & K_{gz}E_{gzx} & K_{gz} \end{array}\right]\left[\begin{array}{c} \omega_{x}\\ \omega_{y}\\ \omega_{z} \end{array}\right]+\left[\begin{array}{c} \omega_{xo}\\ \omega_{yo}\\ \omega_{zo} \end{array}\right]$$

where, *K_gi_*(*i* = *x*, *y*, *z*) denote the FOG scale factors; *E_gij_*(*i*, *j* = *x*, *y*, *z*; *i* ≠ *j*) denote the FOG misalignment errors during installation; *N_gi_*(*i* = *x*, *y*, *z*) denote the FOG output data; ω*_i_*(*i* = *x*, *y*, *z*) denote the turntable alignment axis input angular rate; ω*_io_*(*i* = *x*, *y*, *z*) denote the FOG biases. Therefore, there are 12 static error parameters for tri-axial FOG to be identified in total. 

### 3.2. Derivation of the Optimization Indicator Function

The NAFSA is terminated in one of three conditions. The first is when the maximum number of iterations is reached. The second condition is when the optimization indicator function is below a pre-defined threshold during the optimization process. The third condition is that when performing the next iteration, the deviation of the current iteration result and the next iteration result is within an acceptable range. The optimization indicator is a key factor for the terminate condition during the NAFSA optimization process. The following part presents an optimization indicator function for FOG error parameters identification based on NAFSA.

Theoretically, when the static tri-axial FOG at arbitrary space position, the FOG measured angular rate information would satisfy the following equation:

(12) $$\omega_{x}^{2}+\omega_{y}^{2}+\omega_{z}^{2}=\omega_{ie}^{2}$$

where, ω*_i_*(*i* = *x*, *y*, *z*) are tri-axial FOG theoretical input angular rates; ω*_ie_* = 15.0411°/*h* denotes the Earth rotation angular rate, which is a constant vector along the Earth rotation axis. 

Actually, because of the errors caused by FOG itself, the calculated angular rates are different from theoretical values. Therefore, the angular rate mode square error (MSE) is adopted to represent the deviation, which is derived from Equation (12) and expressed as:

(13) $$\delta \omega ={\hat{\omega }}_{x}^{2}+{\hat{\omega }}_{y}^{2}+{\hat{\omega }}_{z}^{2}-\omega_{ie}^{2}$$

where, ${\hat{\omega }}_{i}(i=x,y,z)$ denote the angular rates calculated from Equation (11) with the stored FOG output data *N_gi_*(*i* = *x*, *y*, *z*).

The target of identifying the steady FOG error coefficients is to make the angular rate MSE as stable as possible. So the standard deviation function is utilized to evaluate the discrete degree of FOG error coefficients:

(14) $$\sigma =\sqrt{\frac{\sum_{j=1}^{M}{\left({\hat{\omega }}_{xj}^{2}+{\hat{\omega }}_{yj}^{2}+{\hat{\omega }}_{zj}^{2}-\omega_{ie}^{2}\right)}^{2}}{M-1}}$$

In Equation (14), *M* denotes the number of positions during optimization process.

### 3.3. MCS-NAFSA Implementation Procedures

The implementation procedures of FOG error coefficients identification are demonstrated in this section. Two main steps are indicated to illustrate the NAFSA optimization process. At the beginning, the variation characteristics of the 12 total error coefficients in the tri-axial FOG are discussed and a clustering process is described with different parameters. After that, the specific MCS-NAFSA FOG error coefficients identification procedures are presented step by step.

#### 3.3.1. FOG Error Coefficients Clustering

In the identification of 12 tri-axial FOG error coefficients, different error coefficients have different influences on the angular rate MSE, and also the NAFSA requires all the AFs to have similar characteristics during the optimization process. Therefore, FOG error coefficients clustering is a necessity before the FOG error coefficients can be optimized by NAFSA.

Thinking about the different error coefficients’ influences on angular rate MSE and based on our previous experiences, the FOG scale factors have the highest impacts on angular rate MSE, followed by the biases, and the last parameters are FOG misalignment errors, so the FOG error coefficients are divided into three categories. They are: three FOG scale factors *K_gi_*(*i* = *x*, *y*, *z*) as category one, three FOG biases ω*_io_*(*i* = *x*, *y*, *z*) as category two, and six FOG misalignment errors *E_gij_*(*i*, *j* = *x*, *y*, *z*; *i* ≠ *j*) as category three. Therefore, when adopting MCS-NAFSA to identify the FOG error parameters, there are three main steps of optimization process should be conducted to implement the highest precision.

#### 3.3.2. MCS-NAFSA FOG Procedures

Through the analysis in Section 3.3.1, within NAFSA FOG procedures, the FOG error coefficients identified by NAFSA are mutually independent in different categories. Hence, three phases of optimization process pseudo-code is shown in the algorithm FOG NAFSA.

In the first phase, FOG scale factor *K_gi_*(*i* = *x*, *y*, *z*) identification is optimized by NAFSA. Firstly, the AFs parameters, category two parameters ω*_io_*(*i* = *x*, *y*, *z*) and category three parameters *E_gij_*(*i*, *j* = *x*, *y*, *z*; *i* ≠ *j*) are all initialized. After that, each AF *i* performs *Individual behavior* and moves to a better position based on the outcome. Subsequently, each AF *i* executes *Group behavior* with respect to their new position. Finally, this process is repeated for N times, and we could calculate the mean value *K_gi___m_*(*i* = *x*, *y*, *z*) as FOG scale factors.

In the second phase, FOG bias ω*_io_*(*i* = *x*, *y*, *z*) identification is indicated by NAFSA. At the beginning, the AFs parameters, category three parameters *E_gij_*(*i*, *j* = *x*, *y*, *z*; *i* ≠ *j*) and optimized FOG scale factors *K_gi___m_*(*i* = *x*, *y*, *z*) are all loaded. Next, all the AFs execute *Individual behavior* and *Group behavior* respectively. At last, this process is repeated for N times, and we could obtain the mean value ω*_io___m_*(*i* = *x*, *y*, *z*) as FOG biases.

In the third phase, FOG misalignment error *E_gij_*(*i*, *j* = *x*, *y*, *z*; *i* ≠ *j*) identification is demonstrated by NAFSA. At first, the AFs parameters, optimized FOG scale factors *K_gi_m_*(*i* = *x*, *y*, *z*) and biases ω*_io_m_*(*i* = *x*, *y*, *z*) are all loaded in initialization process. Second, all the AFs execute *Individual behavior* and *Group behavior*, respectively. Finally, this process is repeated for N times, and we could acquire the mean value *E_gij___m_*(*i*, *j* = *x*, *y*, *z*; *i* ≠ *j*) as FOG misalignment errors. 

Finally, the three stages above are repeated until the FOG error coefficients meet the terminate conditions, when optimization indicator reaches σ < 10^−8^, or the number of iterations reaches a certain preset number.

**Algorithm:** FOG NAFSA.
Begin
    for each AF *i* do
        initialize AFs parameters, category two and category three FOG error parameters
    end
    *bulletin* = arg min *f*(*x_i_*) repeat
        for each AF *i* do
            Perform *Individual behavior*
        end
        for each AF *i* do
            Perform *Group behavior*
        end
    Update *Visual* by Equation (5)
    for each AF *i* do
            initialize AFs and category three FOG error parameters, optimized category one FOG error parameters
    end
    *bulletin* = arg min *f*(*x_i_*) repeat
            for each AF *i* do
               Perform *Individual behavior*
            end
            for each AF *i* do
               Perform *Group behavior*
            end
    Update *Visual* by Equation (5)
    for each AF *i* do
            initialize AFs parameters, optimized category one and category two FOG error parameters
    end
    *bulletin* = arg min *f*(*x_i_*) repeat
            for each AF *i* do
               Perform *Individual behavior*
            end
            for each AF *i* do
               Perform *Group behavior*
            end
    Update *Visual* by Equation (5)
    Until terminate condition meet
End

## 4. Simulation and Discussion

In this section, the FOG error coefficients simulation is conducted by MCS-NAFSA. Before the simulation, the AFs parameters and the non-optimized FOG error parameters at each phase should be preset. Subsequently, the simulation on FOG error parameters is shown by MCS-NAFSA.

### 4.1. Simulation Parameters Preset

The Section 2.3.1 described all the AFs parameters during optimization process. All the preset AFs parameters of tri-axial FOG before the FOG error parameters optimized are listed in Table 1.

**Table 1:** Novel artificial fish swarm algorithm (NAFSA) tri-axial fiber optic gyroscope (FOG) preset artificial fishes (AFs) parameters.

FOG Parameters Types	NAFSA AFs Parameters				
	*Visual*	AFs Numbers	Iteration Times	*CF*_min_	*CF*_max_
*K_gi_*	(5.0000, 2.0000, 0.1000)	50	60	0.000001	0.999999
ω*_io_*	(0.0050, 0.0020, 0.0001)	50	60	0.000001	0.999999
*E_gij_*	(0.0005, 0.0002, 0.00001, 0.000005, 0.000002, 0.000001)	50	60	0.000001	0.999999

Meanwhile, in Section 3.3.2, when one category of FOG error parameters are identified by NAFSA, the other two categories’ parameters also have impacts on angular rate MSE. Therefore, the preset FOG error coefficients are shown in Table 2.

**Table 2:** Preset FOG error coefficients.

Parameters Types	Preset Parameters
$K_{gi}(i=x,y,z)$ (bit h/◦)	$\left[\begin{array}{ccc} 643.50373651 & 645.62852456 & 645.17583651 \end{array}\right]$
$E_{gij}(i,j=x,y,z;i\neq j)$ (°)	$\left[\begin{array}{ccc} 1 & 0.00099183 & 0.00010717\\ \;-0.00037044 & 1 & 0.00014048\\ 0.00010505 & -0.00041586 & 1 \end{array}\right]$
$\omega_{io}(i=x,y,z)$ (°/h)	$\left[\begin{array}{ccc} 0.01562779 & 0.03213767 & 0.02793626 \end{array}\right]$

It is worth noting that, in each of iteration, the dimensions of vector *Visual* listed in Table 1 are equal to the FOG parameter number. So the vector *Visual* dimensions on FOG misalignment errors are different from FOG scale factors and FOG biases. *CF* is a positive number <1, so that its minimum value and maximum value are preset as 0.000001 and 0.999999, respectively. In Table 2, in order to reduce the other two categories’ FOG parameter influences on angular rate MSE during one category of the FOG error parameters optimization process, the preset FOG error parameters are based on the conventional 24-position calibration method [36,37], which is aided by expensive and high-precision turntable in indoor environment.

### 4.2. Simulation Results and Discussion

**Figure 1:**
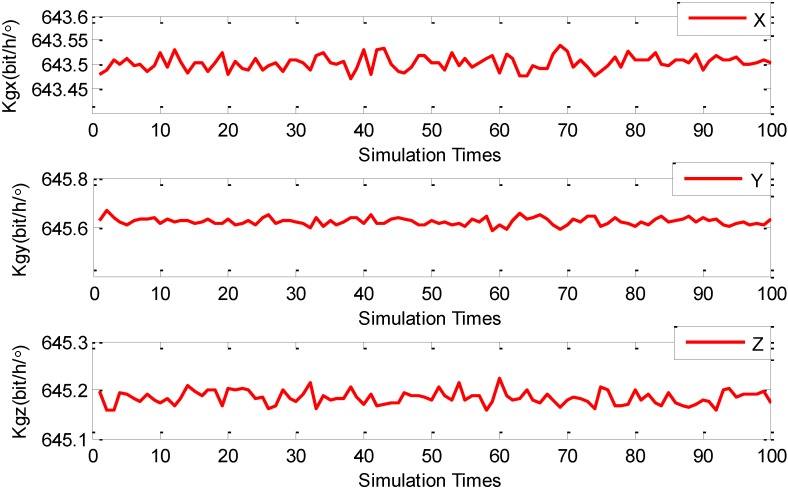
FOG scale factors MCS-NAFSA curves.

**Figure 2:**
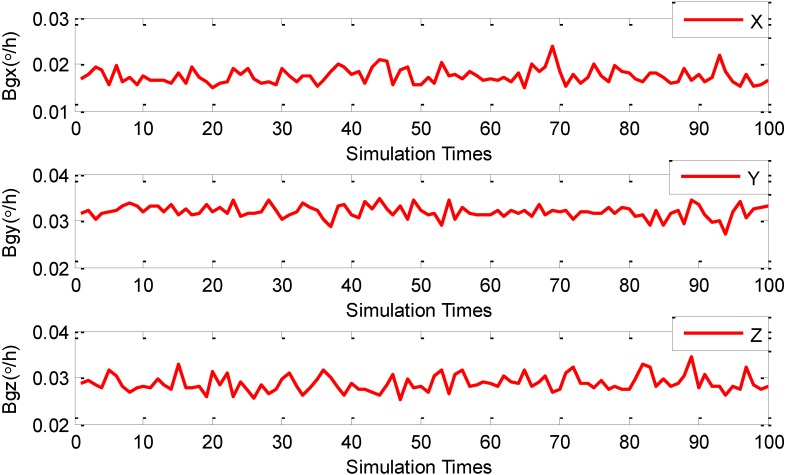
FOG biases MCS-NAFSA curves.

**Figure 3:**
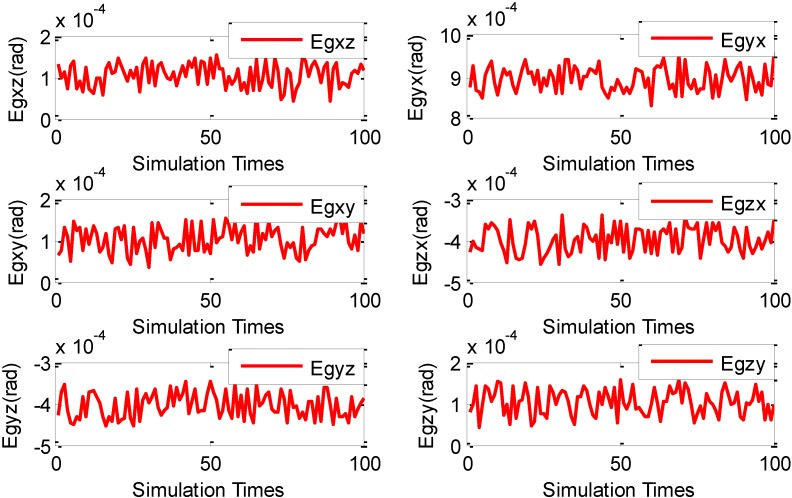
FOG misalignment errors MCS-NAFSA curves.

After the presetting of all the parameters (*i.e.*, the initialization process) is completed, all AFs start to execute the NAFSA optimization procedures. In order to increase the FOG error parameters’ degrees of credibility during the NAFSA optimization process, the random factors are introduced by conducting MCS 100 times after the single NAFSA optimization. Figure 1 shows the three FOG scale factor NAFSA identification curves during the 100 times of MCS. Meanwhile, Figure 2 demonstrates the three FOG bias NAFSA identification curves with 100 times of MCS. Figure 3 presents the six FOG misalignment error NAFSA identification curves with 100 times of MCS.

From Figure 1, Figure 2 and Figure 3, all the FOG error parameters fluctuate during the MCS process within a relatively small range, and the results revealed that the FOG error parameters are influenced by their usage environments. Moreover, the precision of simulation results is also likely to be deteriorated by random factors, such as algorithmic error and computer error, which are easily being neglected and unable to be eliminated during a single NAFSA optimization. So, in this phase, in order to reduce the effects of random factors, the data smoothing method is adopted to calculate the mean value of the MCS results. After being processed by the data smoothing method, all FOG error parameters simulation results are listed in Table 3.

**Table 3:** FOG error parameters MCS-NAFSA identification results.

Parameters	Preset Value	MCS-NAFSA Identification Result	Relative Error	Standard Deviation
$K_{gx}$ (*bit*·*h*/°)	643.50373651	643.50374138	0.00912194	0.01464
$K_{gy}$ (*bit*·*h*/°)	645.62852456	645.62855074	0.04054963	0.01458
$K_{gz}$ (*bit*·*h*/°)	645.17583651	645.17586147	0.03868713	0.01422
$E_{gxz}$ (°)	0.00099183	0.00099161	0.00021812	2.792 × 10^−5^
$E_{gxy}$ (°)	0.00010717	0.00010730	0.00121303	3.155 × 10^−5^
$E_{gyz}$ (°)	−0.00037044	−0.00037021	0.00062088	3.069 × 10^−5^
$E_{gyx}$ (°)	0.00014048	0.00014061	0.00092540	2.924 × 10^−5^
$E_{gzy}$ (°)	0.00010505	0.00010533	0.00247501	3.026 × 10^−5^
$E_{gzx}$ (°)	−0.00041586	−0.00041614	0.00067330	3.176 × 10^−5^
$\omega_{xo}$ (°/ *h*)	0.01562779	0.01565329	0.001633171	0.001632
$\omega_{yo}$ (°/ *h*)	0.03213767	0.03217083	0.001031810	0.001369
$\omega_{zo}$ (°/ *h*)	0.02793626	0.02790215	0.001222490	0.001780

Through the comparison between the preset FOG error parameters and the NAFSA identification results in Table 3, we can summarize that the relative error amplitudes of each parameter are substantially small and the credibility of identification results is enough for the FOG-based SINS navigation requirements. FOG scale factors *K_gi_*(*i* = *x*, *y*, *z*) relative error magnitudes are small enough at 10^−2^~10^−3^ ppm, to meet the high-precision navigation requirements completely. The relative errors of FOG biases ω*_io_*(*i* = *x*, *y*, *z*) and misalignment errors *E_gij_*(*i*, *j* = *x*, *y*, *z*; *i* ≠ *j*) could also reach 10^−3^~10^−4^ in magnitudes which are also completely enough to satisfy the FOG-based SINS precision demands. However, in OAFSA, the FOG scale factors relative error magnitudes are 10^−1^~10^−2^ ppm and the FOG biases and misalignment errors’ relative error magnitudes are 10^−2^~10^−3^ [29,30]. Therefore, theoretically, the precision of NAFSA identification results is an order of magnitude higher than OAFSA in FOG error parameters identification.

Moreover, the standard deviations of the estimates are indicators to show the stability of the estimates [38,39]. In Table 3, the standard deviations of the FOG scale factors *K_gi_*(*i* = *x*, *y*, *z*) are 0.01464(*bit*·*h*/°), 0.01458(*bit*·*h*/°) and 0.01422(*bit*·*h*/°), respectively. The standard deviations of the FOG misalignment errors *E_gij_*(*i*, *j* = *x*, *y*, *z*; *i* ≠ *j*) are 2.792 × 10^−5^ (°), 3.155 × 10^−5^ (°), 3.069 × 10^−5^ (°), 2.924 × 10^−5^ (°), 3.026 × 10^−5^ (°) and 3.176 × 10^−5^ (°). Moreover, the standard deviations for the FOG biases errors ω*_io_*(*i* = *x*, *y*, *z*) are 0.001632(°/*h*), 0.001369(°/*h*) and 0.001780(°/*h*) on each axis. From the standard deviations of these three categories of FOG error parameters, the standard deviations of FOG scale factors are greater than FOG biases, and the standard deviations of FOG biases are also greater than FOG misalignment errors. This phenomenon corresponds to the FOG error coefficients clustering principle in the Section 3.3.1. More importantly, the estimated FOG error parameters could satisfy the preset optimization indicator in Equation (14).

**Figure 4:**
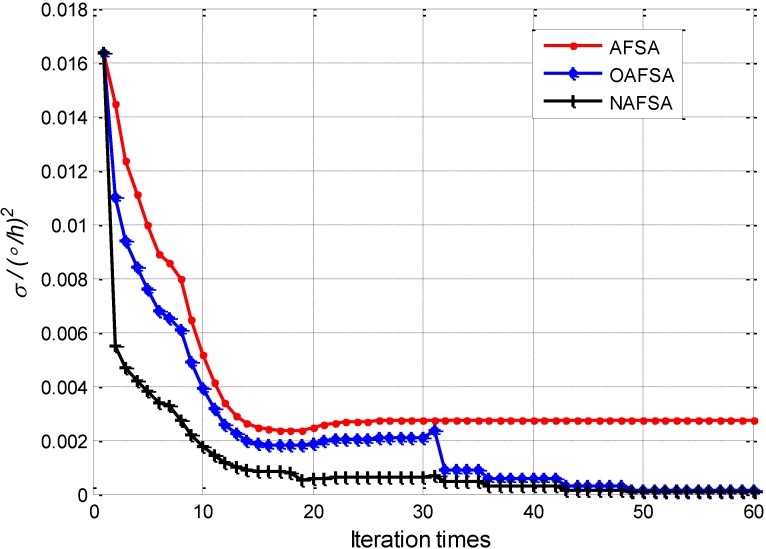
Indicator functions of SAFSA, OAFSA and NAFSA.

Furthermore, the variation tendencies of indicator functions among the SAFSA, OAFSA and NAFSA, when they are used for identifying the FOG scale factors, are demonstrated in Figure 4.

In Figure 4, the red dotted curve denotes the SAFSA indicator function variation tendency. After 20 iterations, the indicator function has a slight increase in tendency, and it remains stable after 24 iterations, with the indicator function reaching 0.002584(°/*h*)^2^. However, the OAFSA’s indicator function with the blue star curve has a faster convergence rate than SAFSA, but the indicator function begins to diverge after 20 iterations. The secondary initialization method is adopted in 30 iterations to decrease the indicator function and to improve the convergence precision. For comparison, the NAFSA optimization indicator function is shown with the black plus curve in Figure 4. We can conclude that the NAFSA has a better convergence rate than the OAFSA and SAFSA because of the reduction of the algorithm’s structural and computational complexities. Moreover, it is evident that the NAFSA indicator function is always convergent during the optimization progress, which is due to the usage of the previous experience of AF. Therefore, the NAFSA has better performance in convergence rates and reliability of the optimized results compared to SAFSA and OAFSA.

## 5. Experiments and Discussion

To validate the feasibility and priorities of the NAFSA on FOG error parameters optimization, the static and dynamic navigation experiments were conducted, respectively. Before these two experiments, the FOG error parameters were calibrated by using a turntable with 24-position method. The navigation information output results are also based on the FOG error parameters that are calibrated by this 24-position method. Additionally, for comparison with OAFSA and NAFSA in navigation experiments, the stored experimental data were also used for navigation mechanization with FOG error parameters identified by OAFSA and NAFSA.

**Figure 5:**
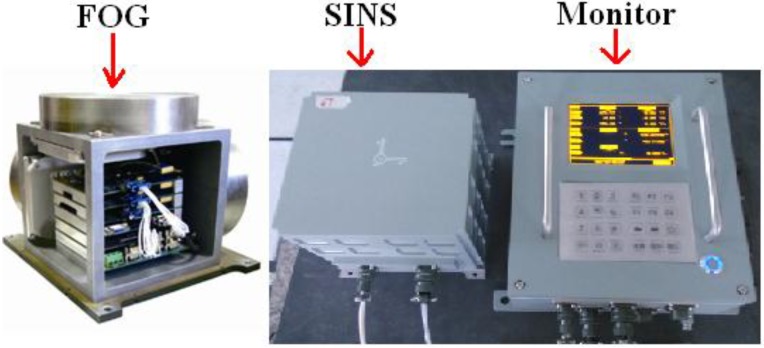
The FOG and FOG-based SINS.

For both experiments, the FOG-based SINS was developed by Inertial Navigation and Measurement & Control Technology Institute at Harbin Engineering University. The main performance indicators of FOG are demonstrated in Table 4. Figure 5 shows the FOG and the FOG-based SINS in experiments.

**Table 4:** FOG performance indicators.

Parameter Items	Performance Indicators
FOG dynamic range (°/s)	−800–+800
FOG scale factor stability (ppm)	10
FOG bias instability (°/h)	0.005
FOG angular random walk (°/h^1/2^)	0.0005

### 5.1. Static Navigation Experiment and Discussion

#### 5.1.1. Experimental Procedures and Data Processing

In this section, a static navigation experiment is carried out. At the beginning, the FOG-based SINS and the corresponding monitor are installed on the marble benchmark that is used to eliminate external disturbances on system positioning precision. Then, the SINS is started, the turntable calibrated FOG error parameters and the initial navigation information (initial position and velocity) are loaded. After that, both the inertial measurement unit (IMU) output and the navigation information for 24 h are stored after the SINS completes the initial alignment process.

After obtaining the stored 24 h IMU output and navigation information, we first used the FOG data to recalibrate the FOG error parameters by OAFSA and NAFSA, respectively. Second, the navigation mechanization process was conducted again with the FOG error parameters optimized by OAFSA and NAFSA, respectively. Finally, the positioning error curves were plotted and the positioning error numerical results were obtained with the three methods introduced.

The positioning error is calculated by [40,41]:

(15) $$P_{error}=\frac{\sqrt{{\left(lat-lat_{0}\right)}^{2}*R^{2}+{\left(long-long_{0}\right)}^{2}*{\left(R*\cos(lat)\right)}^{2}}}{1851.8518}$$

where, *long*_0_ and *lat*_0_ are the initial longitude and latitude of the SINS, and *long* and *lat* are the calculated longitude and latitude. *R* denotes the radius of Earth.

#### 5.1.2. Experimental Results and Discussion

**Figure 6:**
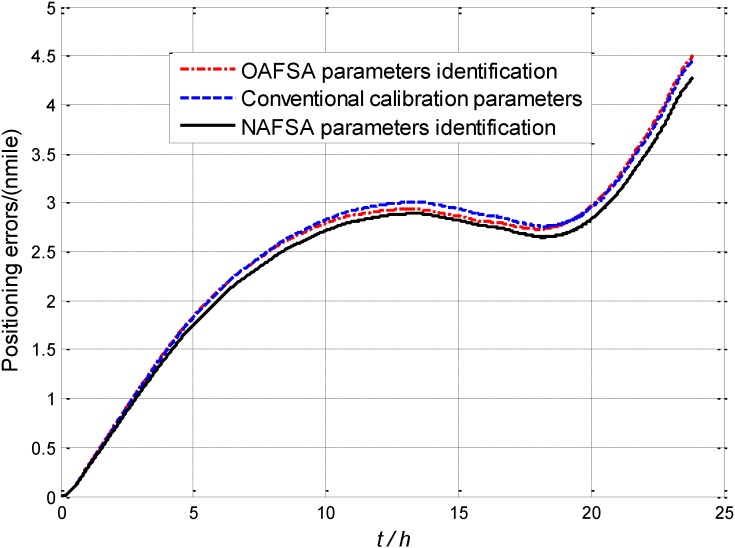
The comparison of positioning error with three methods.

Figure 6 shows a comparison of positioning errors in 24 h static navigation experiment when the FOG error parameters are identified by the conventional calibration method, OAFSA and NAFSA, respectively. The red dotted positioning error curve represents the OAFSA FOG error parameters identification results. Additionally, the blue dotted curve represents the positioning error curve with FOG calibrated by conventional high-precision turntable method. Both curves present positioning precision of 4.5 nautical miles in 24 h static navigation, which shows that the OAFSA could substitute the conventional calibration method without using high-precision turntable [29,30]. Moreover, it is worth noting that the black solid curve in Figure 6 denotes positioning precision of the NAFSA on FOG error parameters identification. The curve’s tendency demonstrated that after 5 h of navigation, the positioning error is lower than the other two methods and the precision is about 0.3 nautical miles better than the OAFSA in one day of navigation.

The corresponding numerical results of static positioning errors with the three different methods are shown in Table 5. Both the conventional calibrated and the OAFSA recalibrated FOG-based SINS have about 4.5 nautical miles positioning error in 24 h. Meanwhile, the NAFSA recalibrated FOG-based SINS has 4.255 nautical miles of positioning error. Therefore, the static navigation experiment demonstrates that the NAFSA recalibrated FOG-based SINS is superior to that of the conventional calibrated and the OAFSA recalibrated FOG-based SINS.

**Table 5:** Static positioning results of three different methods.

Methods	Conventional Calibration Method	OAFSA Identification Method	NAFSA Identification Method
24 h positioning error (nautical mile)	4.4895	4.4988	4.2550

### 5.2. Dynamic Navigation Experiment and Discussion

#### 5.2.1. Experimental Procedures and Data Processing

**Figure 7:**
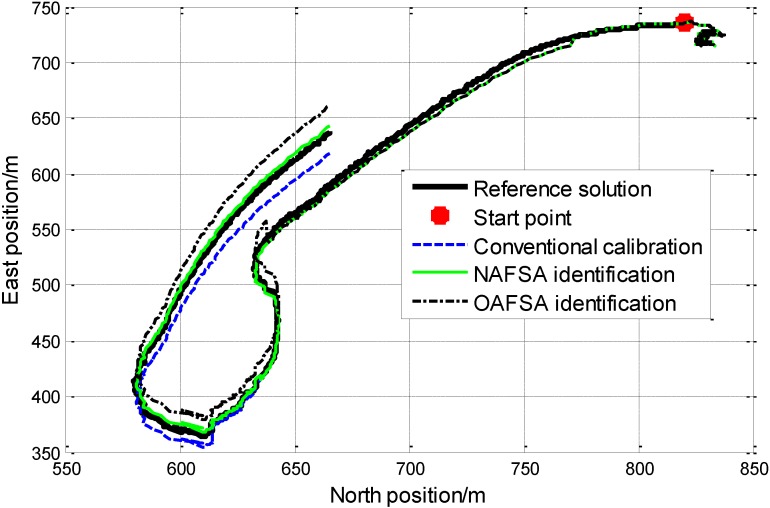
Trajectory comparison between different methods.

In order to validate the feasibility and priorities of the NAFSA in real application conditions, a lake navigation experiment was also conducted in Qiandao Lake for a period of time. Firstly, the FOG-based SINS and the reference system, the difference global positioning system (DGPS) receiver, were installed in a ship. Secondly, after the FOG-based SINS finished the mooring alignment process at the starting point, the ship sailed successively with speed change, heading change, manoeuvres, *etc.* At the same time, the reference DGPS information, IMU data and the self-developed FOG-based SINS navigation information were all collected and saved. Finally, the data was processed the same way as Section 5.1.1. The trajectories of the lake experiment with GPS and the SINS when FOG parameters are identified by three different ways are all demonstrated in Figure 7. Moreover, the numerical results of the system positioning errors in both the North and East directions are calculated and listed in Table 6.

**Table 6:** Dynamic positioning errors of three different methods.

Methods	Conventional Calibration Method	OAFSA Identification Method	NAFSA Identification Method
North direction positioning error (m)	5.1154	5.2131	5.0134
East direction positioning error (m)	20.0253	20.3580	8.1689

#### 5.2.2. Experimental Results and Discussion

On one hand, Figure 7, shows that the conventional calibration method, the OAFSA identification method and the NAFSA identification method on FOG error parameters all have the ability to implement the FOG error parameters calculation and reach different degrees of positioning precision in the lake experiment. On the other hand, the green curve shows that the NAFSA recalibration method is superior, such as better robustness when speed and heading change, better tracking capability during the whole navigation process, which means higher positioning precision. Moreover, by utilizing the NAFSA on FOG error parameters identification, some lower precision SINS would have better performance for parameters identification after a specific period of navigation.

The lake experiment positioning errors compared with reference solution at the end of the navigation are listed in Table 6. We found that the conventional calibration method and the OAFSA identification method have almost the same positioning errors. The conventional calibration method has a North direction positioning error around 5.1154 m and East direction error about 20.0253 m. The OAFSA identification method has a North direction positioning error around 5.2131 m and an East direction error about 20.3580 m. While the NAFSA identification method has better performance in terms of positioning error, with a North direction positioning error of 5.0134 m, and East direction error of 8.1689 m. By comparing the North direction positioning errors with these three methods, the NAFSA method has only a slightly smaller positioning error. Furthermore, the NAFSA recalibration method could improve the East direction positioning error of the conventional calibration and OAFSA recalibration methods from about 20 m to 8.169 m, which could clearly demonstrate the priorities of the NAFSA recalibration method. Therefore, the NAFSA recalibration method is a more powerful choice in its engineering application for FOG error parameters recalibration.

All in all, in both experiments, the NAFSA recalibration method has advantages in workload and costs compared to the conventional calibration method. However, it presents better performance in long-term navigation precision and is more acceptable for actual engineering applications than previous OAFSA recalibration methods, which is mainly due to the lower structural and computational complexities and faster convergence rate of the NAFSA recalibration method.

## 6. Conclusions

After the FOG-based SINS operated for a period of time, the FOG would be vulnerable to the working environmental disturbances, such as gravitational field, magnetic field and thermal field, which cause nonreciprocal phase shifts except for the rotary movement by the vehicle itself. These exterior disturbances could influence the FOG error parameters’ stability directly or indirectly. Even though some advanced measures are taken to eliminate these effects, high-precision navigation application is far from enough.

This research work is based on one of the swarm intelligence algorithms, NAFSA, focusing mainly on its combination with MCS and utilization in FOG error parameters identification. The NAFSA has the advantages of lower structural and computational complexities and higher convergence rates than the previous OAFSA recalibration method during the optimization process. It also has lesser workload and costs requirements than the conventional FOG error parameters calibration methods. Furthermore, the non-autonomous property could be avoided when compared with the previous OAFSA recalibration method. Therefore, the NAFSA FOG error parameters recalibration method could implement longer recalibration interval time with higher precision in some harness application environments.

When the FOG-based SINS applied in navigation conditions, NAFSA-identified FOG error parameters could realize the SINS navigation process rapidly and accurately. Moreover, the NAFSA-identified FOG error parameters have better environmental adaptive ability, which means higher positioning accuracy and better tracking performance. Therefore, the NAFSA recalibration method has better ability than the conventional calibration method and the previous OAFSA in FOG error parameters recalibration application.

However, the AFSA on FOG error parameters recalibration is only in an exploratory phase and all the navigation experiments are based on the stored data. Thus, our work for the next stage is to realize the algorithm in real-time navigation.
